# Antipsoriatic Effects of Wannachawee Recipe on Imiquimod-Induced Psoriasis-Like Dermatitis in BALB/c Mice

**DOI:** 10.1155/2018/7931031

**Published:** 2018-01-24

**Authors:** Mingkwan Na Takuathung, Ariyaphong Wongnoppavich, Ampai Panthong, Parirat Khonsung, Natthakarn Chiranthanut, Noppamas Soonthornchareonnon, Seewaboon Sireeratawong

**Affiliations:** ^1^Department of Pharmacology, Faculty of Medicine and Graduate School, Chiang Mai University, Muang 50200, Thailand; ^2^Department of Biochemistry, Faculty of Medicine, Chiang Mai University, Muang, Chiang Mai 50200, Thailand; ^3^Department of Pharmacology, Faculty of Medicine, Chiang Mai University, Muang, Chiang Mai 50200, Thailand; ^4^Department of Pharmacognosy, Faculty of Pharmacy, Mahidol University, Rajathevi, Bangkok 10400, Thailand

## Abstract

Psoriasis is a common immune-mediated chronic inflammatory skin disease characterized by thick and erythema raised plaques with adherent silvery scales. T-cells are activated via the IL-23/Th17 axis which is involved in psoriasis pathogenesis. Conventional treatments of psoriasis have adverse events that influence patients' adherence. Wannachawee Recipe (WCR) is Thai traditional medicine that is known to be effective for psoriasis patients; however, preclinical evidence is still lacking. This study investigated the therapeutic potential of WCR on antiproliferant activity using imiquimod- (IMQ-) induced psoriasis-like dermatitis in a mouse model. Psoriasis-like dermatitis was induced on the shaved dorsal skin and right ear pinna of BALB/c mice by topical application of IMQ for 15 consecutive days after which WCR was administered to the mice by oral gavage for 10 days. Phenotypical observations, histopathological examinations, and ELISA of skin and blood samples were conducted. WCR significantly ameliorated development of IMQ-induced psoriasis-like dermatitis and reduced levels of Th17 cytokines (IL-17A, IL-22, and IL-23) in both serum and dorsal skin. Histopathological findings showed a decrease in epidermal thickness and inflammatory T-cell infiltration in the WCR-treated groups. The WCR has pharmacological actions which regulate Th17 related cytokines suggesting that it is a potential alternative therapeutic strategy for psoriasis.

## 1. Background

Psoriasis, a noncommunicable and immune-mediated inflammatory skin disorder, is characterized by sharply demarcated, red, scaly plaques most often on the elbows, knees, scalp, and lumbar area [1]. Symptoms frequently reported by patients include scaling, itching, erythema, burning, and bleeding [2]. Comorbid diseases of psoriasis (e.g., Crohn's disease, metabolic syndrome, cancer, depression, and cardiovascular diseases) further increase the physical and psychological burden [3]. Strong evidence exists that the cell-mediated adaptive immune system, T helper 17 (Th17), plays critical roles in psoriasis, while myeloid cell-produced interleukin-23 (IL-23) functions as a key cytokine for the expansion and maintenance of Th17 cells [4]. Th17 cells and their downstream effector molecules, which include IL-17A, IL-22, and TNF-*α*, have been shown to induce keratinocyte proliferation and other hallmark features of psoriasis [5].

Conventional therapies such as corticosteroids, vitamin D3 analogues, and calcineurin inhibitors are currently used as topical therapies in mild psoriasis. Severe psoriasis often necessitates treatment with phototherapy or systemic agents including methotrexate, cyclosporine, and acitretin [6]. Biologic therapies that act on the upregulated cytokine pathways have also been developed and approved for psoriasis treatment [7]. However, most of these regimens have well-documented arrays of adverse effects that seem to be the main factor hampering patients' adherence to long-term psoriasis treatment [8], suggesting a need for development of a drug that would provide improved effectiveness but with fewer side effects. Traditional medicine, which provides front-line pharmacotherapy for billions of people worldwide, represents a possible source of a solution, although the Western medical establishment is often skeptical about its efficacy due in part to a general lack of preclinical and clinical evidence [9].

Traditional systems of medicine in Thailand employ many different plants for the treatment of dermatological conditions [10]. Wannachawee Recipe (WCR) has been enlisted in the Hospital Traditional Medicine Formulary and has been used as a Thai medicine for the treatment of psoriasis in the Thai Traditional Medicine Clinic of Prapokklao Hospital since 2006. An observational study conducted at that hospital indicated that psoriasis patients aged between 13 and 97 years old with different severity of psoriasis attended at this clinic. All patients received WCR 10.77 g per day for 6-7 months. The results demonstrated that 92.80% of 136 Thai psoriasis patients had good responses with WCR, whereas 7.21% of them showed less improvement [11]. This observation might be from the fact that the patients were recently provoked by triggering factors such as chemical and sunlight exposure, stress, and sleep deprivation [11]. A previous study by Na Takuathung et al. demonstrates that WCR can inhibit the growth and viability of keratinocytes. In addition, WCR significantly decreases the gene expression of IL-1*β*, IL-6, IL-8, IL-17A, IL-22, IL-23, and TNF-*α* as well as significantly decreasing the secretion of IL-17A, IL-22, and IL-23 in TNF-*α*- and IFN-*γ*-induced HaCaT cells [12]. Although the WCR has been clinically used as a clinical therapy at the Thai Traditional Medicine Clinic for many years and it was proved to have potent anti-inflammatory effects on the HaCaT cells, there is still a lack of supporting* in vivo* information regarding pharmacological actions of the WCR in psoriasis treatment.

For the experimental design, we used imiquimod (IMQ) for induction of psoriasis-like dermatitis. IMQ activates the toll-like receptor-7/8 (TLR-7/8), which is used to treat genital warts in patients [13]. IMQ-induced psoriasis-like dermatitis in BALB/c mice is mediated via the IL-23/IL-17 axis. This mouse model has been described as closely resembling human plaque-type psoriasis [14]. The present study used an IMQ-induced psoriasis-like dermatitis models to examine the potential therapeutic antiproliferant activity of the WCR to develop preclinical evidence of its efficacy in psoriasis therapy.

## 2. Materials and Methods

### 2.1. Chemicals and Reagents

Unless stated, all chemicals and reagents were purchased from Sigma Aldrich Co. (Merck KGaA, Darmstadt, Germany). Acitretin 25 mg was purchased from Silom Medical Co., Ltd. (Thailand), and IMQ cream (Aldara) from 3M Pharmaceuticals. The rodent diet CPF 082 was obtained from CP MICE FEED, SWT Co., Ltd., Samut Prakan, Thailand, and sterile disposable plastic needles were obtained from Strategic Applications, Inc., USA.

### 2.2. Drug Preparation

WCR is composed of 8 Thai herbs as shown in Table 1. The dosage used in the present study was determined based on dosages prescribed at Prapokklao Hospital, Chanthaburi Province, Thailand. Each medicinal plant was obtained from the provinces in Thailand as previously described [12]. All herbal plants were preliminarily examined by qualified Thai traditional doctors. Qualitative and quantitative analysis was performed for both herbal plants and WCR following the methods in Thai Herbal Pharmacopoeias and WHO guideline [15, 16], by Associate Professor Dr. Noppamas Soonthornchareonnon, Mahidol University, Bangkok, Thailand.

WCR was prepared as previously described [12]. Briefly, each crude herb was dried in a hot air oven; then it was grounded and sieved. The mixture of crude components of WCR in distilled water was refluxed; then the extract was percolated through a filter cloth. The WCR extract was concentrated by a rotary evaporator and dried by spray apparatus. The yield of WCR was 11.77% of the dried weight.

### 2.3. Animals

Eight- to eleven-week-old BALB/cMlac male mice* (Mus musculus)* weighing approximately 20 g each were purchased from the National Laboratory Animal Center, Mahidol University, Thailand. The animals were housed under strict hygienic conventional conditions in cages with corncob bedding material at 21 ± 1°C with 50–70% relative humidity and were subjected to a 12 h light/12 h dark cycle in the Laboratory Animal House of the Faculty of Medicine, Chiang Mai University, Thailand. The size of cages was 206 × 365 × 140 mm, which contained 7 mice per cage. The mice were acclimatized for 1 week and were provided with commercial rodent diet CPF 082 (082 CP MICE FEED, SWT Co., Ltd., Samut Prakan, Thailand) and water ad libitum before being used in the research. Prior to and during the experimental period, all mice were assessed for their health status, including food and water intake, bodyweight, behavioral signs, respiratory patterns, and cardiovascular signs. Experiments were conducted according to international and national guidelines for ethical conduct in the care and use of animals and were approved by the Animal Ethics Committee of the Faculty of Medicine, Chiang Mai University (Protocol approval number: 14/2559).

**Figure 1:**
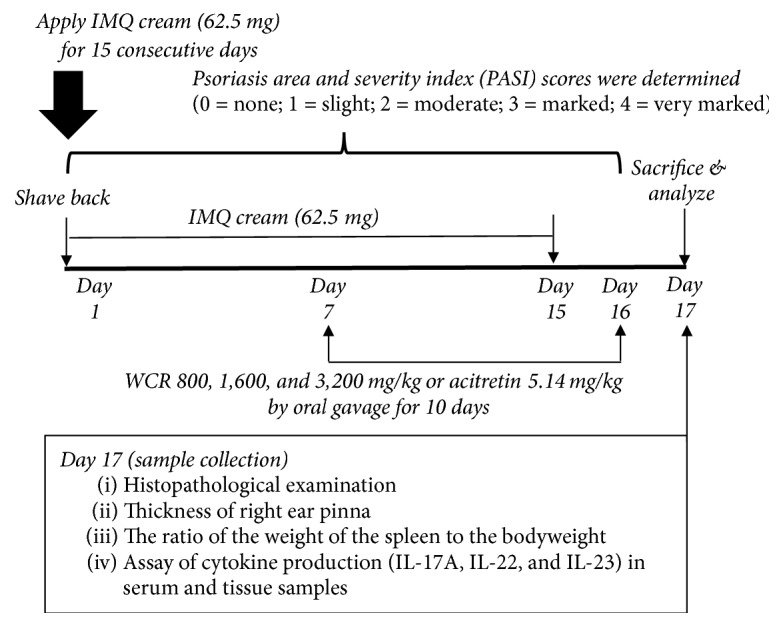
Experimental procedure to induce psoriasis-like dermatitis in mice group II to group VI. IMQ, imiquimod; WCR, Wannachawee Recipe.

Histological and clinical features of dermatitis in mice induced by IMQ are similar to psoriasis manifestations, including erythema, scaling, and inflammation [14]. The dorsal skin of the mice was shaved and a commercially available 5% IMQ cream (Aldara, 3M Pharmaceuticals) was applied. The sample size of mice was calculated using *G*^*∗*^Power software [17, 18]. The mice were randomly divided into 6 groups of 7 animals and received the treatment as shown in Figure 1. Briefly, on day 1 and continuing for 15 consecutive days, mice in all groups except for the normal group received a daily topical dose of 62.50 mg of the IMQ cream on the shaved area of their backs [19]. This translates into a daily dose of 3.125 mg of the active ingredient. On the right ear, 5% IMQ cream was applied at a dose of 20 mg/cm^2^ [20]. A test substance (WCR) and a standard reference (acitretin) were freshly prepared in water before being given to the mice [21]. Mice received the treatment once a day in the morning for 10 days by oral gavage (sterile disposable plastic needles, Strategic Applications, Inc., USA).

  Group I: normal (negative control) group received only a Vaseline® on shaved dorsal skin surface and right ear pinna.
  Group II: positive control group received a daily topical dose of 5% IMQ cream on shaved back and right ear pinna (left ear pinna was untreated).
  Group III: standard group received IMQ plus acitretin at 5.14 mg/kg once daily by oral gavage.
  Group IV: sample group received IMQ plus WCR 800 mg/kg once daily by oral gavage.
  Group V: sample group received IMQ plus WCR 1,600 mg/kg once daily by oral gavage.
  Group VI: sample group received IMQ plus WCR 3,200 mg/kg once daily by oral gavage.

### 2.4. Sample Collection

All the mice in groups I to VI were sacrificed at the end of the experiment by an intraperitoneal injected overdose (50–90 mg/kg) of pentobarbital (Nembutal®; Ceva Santé Animale, France). Pentobarbital was used in this study because its profile on the sleeping time and loss of righting reflex has been well established after pentobarbital administration [22]. Mice in groups II to VI received IMQ treatment for 15 days and either WCR or acitretin treatment from day 7 through day 16. They were then sacrificed on day 17 at the laboratory. Central dorsal skin tissue (approximately 1 cm^2^) and right ear pinna from all the groups were excised for histological studies [23] and cytokine production analysis [20]. Blood samples from all the groups were collected in clean dry vials for cytokine production analysis [23].

### 2.5. Scoring Severity of Skin Inflammation

Psoriasis area and severity index (PASI) scores were determined by evaluating the degree of erythema, thickening, and scaling on the affected dorsal skin surface and ear pinna. PASI for each was measured on a four-point scale (0 = none; 1 = slight; 2 = moderate; 3 = marked; 4 = very marked). The severity of skin inflammation was measured by the combined scores (erythema plus scaling plus thickening) giving a range of scores of 0–12. Ear thickness was measured twice every other day using digital calipers (BEC, China). An increase in ear thickness was used to indicate the extent of epidermal proliferation and inflammation.

### 2.6. Histopathological and Immunohistochemical Examinations

Selected tissue samples were fixed in 10% neutral-buffered formalin before processing and embedding in paraffin blocks. Sections of the samples were prepared at 4 *μ*m thickness using a rotary microtome and were stained with hematoxylin and eosin (H&E) stain; then they were observed under a microscope using a digital camera system.

### 2.7. Changes of Spleen in Mice

The spleen from each mouse was isolated and a photograph was taken before being weighed. Splenomegaly was evaluated by calculating the ratio of the weight of the spleen to the bodyweight [24].

### 2.8. Assay of Cytokines Production

To measure cytokine levels in serum, mouse blood was collected at 24 h after the final treatment using the cardiac puncture method; serum was stored at −70°C until analysis. To measure cytokine levels in skin tissue, the central dorsal skins of the mice were removed and stored at −80°C. The skins were later homogenized in tissue protein lysis buffer (Bio Basic Inc., Canada) at 4°C, and the supernatants were stored at −80°C until analysis. The concentrations of IL-17A, IL-22, and IL-23 in the mouse serum and skin tissue were measured using mouse IL-17A, IL-22, and IL-23 ELISA MAX™ Deluxe (BioLegend, USA). ELISA was performed in accordance with the manufacturer's instructions.

### 2.9. Statistical Analysis

Differences between treatment groups were analyzed by one-way analysis of variance (one-way ANOVA), followed by post hoc Fisher's least significant difference (LSD) test using the SPSS for Windows (version 20.0). Differences were considered significant at *P* < 0.05. All experiments were performed in triplicate using a minimum of three replicates. All values are expressed as mean ± standard deviation (SD).

## 3. Results

### 3.1. Effect of IMQ-Induced Psoriasis-Like Dermatitis in Mice

**Figure 2:**
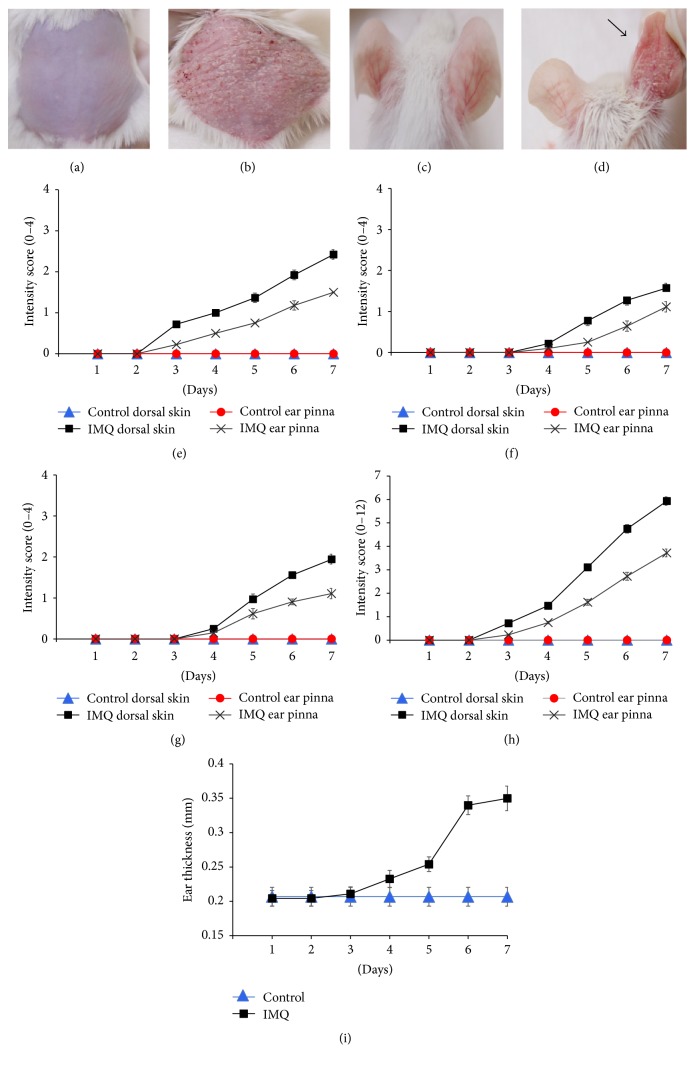
Phenotypical observations of dorsal skin and ear pinna in treated mice groups. (a) Control mice (group I) with daily topical application of Vaseline on the shaved dorsal skin and ear pinna. (b) Test mice with daily IMQ-treated (5%) dorsal skin (group II) on day 7 after IMQ treatment showing psoriasis-like inflammation and erythema lesions on treated dorsal skin. (c) Ear pinna of the control mice. (d) IMQ-treated inflamed right ear pinna (marked with* arrow*) and the untreated left ear pinna of the same mouse. PASI scores showing intensity of (e) erythema, (f) thickness, and (g) scales of the control and treated mice dorsal skin and right ear pinna on a 0–4 point scale (none to maximum). (h) Cumulative score (erythema plus thickness plus scaling). Figures are mean score ± SD of the seven mice in each group. (i) Ear thickness of the right ear pinna. Mean ear thickness ± SD of the seven mice in each group.

Characteristics and health status such as food and water consumption, bodyweight, behavior signs, respiratory patterns, and cardiovascular signs of all mice were normal throughout the experimental period. Two or three days after starting IMQ application, it was observed that both the dorsal skin and the right ear pinna of the mice exhibited signs of erythema, scaling, and thickening (Figures 2(b) and 2(d)). Thereafter, the intensity of psoriasis-like symptoms of group II mice progressively increased in severity till the end of the treatment (day 16). However, mice in group I treated daily with Vaseline did not show any signs of inflammation on dorsal skin or right ear pinna (Figures 2(a) and 2(c)). The independent PASI scores depicted in Figures 2(e)–2(h) show the continually increasing levels of inflammation after IMQ application from day 1 to day 7, before initiation of either acitretin or WCR treatment. The PASI scores reached peak intensity at the seventh day after IMQ treatment which indicates successful induction of psoriasis-like dermatitis in the IMQ-treated mice. Ear thickness of the mice was measured as an independent parameter of skin inflammation.  Figure 2(i) shows a significant increase in ear thickness of IMQ-treated mice compared with control mice from day 5 or day 6 onward.

### 3.2. Phenotypical Observations of WCR-Treated IMQ-Induced Psoriasis-Like Dermatitis in Mice

**Figure 3:**
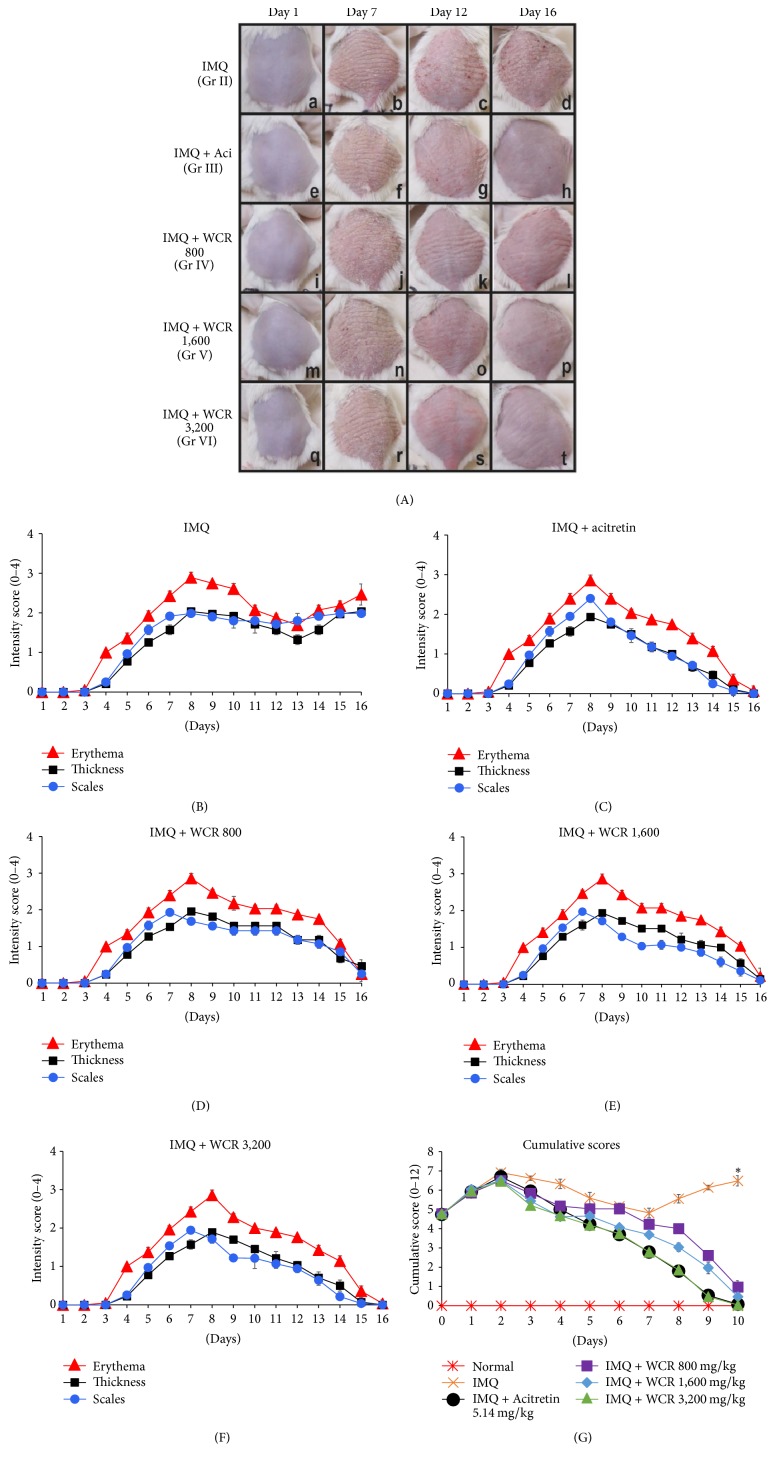
Phenotypical presentation of the effect of WCR treatment on the dorsal skin of IMQ-induced psoriasis-like dermatitis. (A) Phenotypical presentation of dorsal skin of the five dermatitis groups; group II (IMQ, (a–d)), group III (acitretin 5.14 mg/kg + IMQ, (e–h)), group IV (WCR 800 mg/kg + IMQ, (i–l)), group V (WCR 1,600 mg/kg + IMQ, (m–p)), and group VI (WCR 3,200 mg/kg + IMQ, (q–t)) on day 1 (no treatment), day 7 (after IMQ treatment, psoriasis-like dermatitis-induced mice), day 12 (acitretin- or WCR-treated psoriasis-like dermatitis-induced mice), and day 16 (end of acitretin or WCR treatment). (B–F) PASI intensity scores (erythema, thickness, and scales) recorded on days 1–16 for mice groups II–VI. (G) PASI cumulative scores recorded for 10 days after administration of acitretin or WCR. ^*∗*^Statistically significant at *P* < 0.001 compared with all other groups. Grp, group; Aci, acitretin; WCR, Wannachawee Recipe; and IMQ, imiquimod.

Analysis of phenotypical presentations of WCR-treated IMQ-induced inflammation accompanied by structural feature characteristic of psoriasis found that dorsal skin started to display erythema, thickening, and scaling beginning 2 to 3 days after the first IMQ application, with maximum inflammatory severity occurring at days 7 and 8. Intensity of the psoriasis-like symptoms in the IMQ-only treated group (group II) was observed to have steadily increased from day 1 through day 16 (Figure 3(A)(a–d)). However, there was a statistically significant decrease in psoriasis-like symptoms beginning at day 9, the second day after initiation of treatment with either acitretin (group III) or WCR (groups IV–VI). These symptoms consistently declined until day 16, the end of the acitretin or WCR treatment (Figure 3(A)(e–t)). The individual PASI scores and the cumulative scores of all groups from days 1 to 16 are depicted in Figures 3(B)–3(G). Comparing with the IMQ-treated group, all WCR-treated groups showed a significant inhibitory effect on IMQ-induced psoriasis-like dermatitis. The dose-dependent reduction in the PASI inflammatory symptoms was observed in the WCR-treated group at dose administrations of 800, 1,600, and 3,200 mg/kg body weight of mice. The marked reduction of PASI scores of acitretin-treated group was comparable to the 3,200 mg/kg WCR-treated group.

### 3.3. Histopathological Analysis

**Figure 4:**
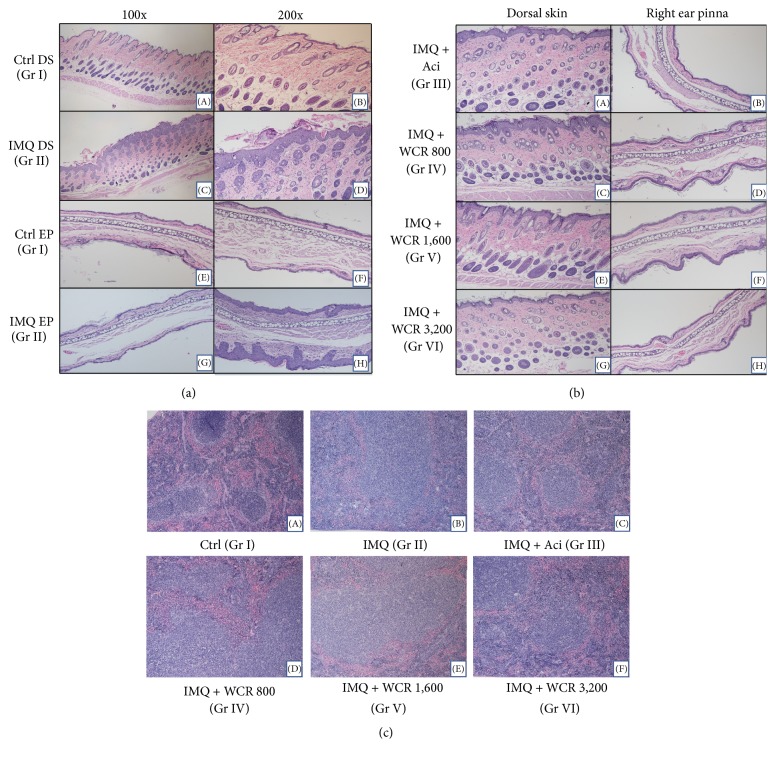
Histological examinations of the six different treatment groups, stained with hematoxylin and eosin (H&E). (a) H&E stained dorsal skin and right ear pinna of control and IMQ-treated mice. Images magnified 100x (A, C, E, and G) and 200x (B, D, F, and H). (A, B) Control dorsal skin tissue section treated with Vaseline showing normal epidermis, dermis, sebaceous glands, and hair follicles. (C, D) Dorsal skin tissue section of IMQ-treated mice showing flaky crust, acanthosis, and hyperkeratosis of the epidermis. Abundant inflammatory infiltration and elongated rete ridges were also shown with IMQ treatment. (E, F) Control group ear pinna section showing normal epidermis, dermis, and cartilage. (G, H) Right ear pinna of IMQ-treated mice showing epidermal hyperplasia and disturbance in the normal pattern of the dermal layer. (b) Histopathology of acitretin- and WCR-treated dorsal skin sections (A, C, E, and G) and right ear pinna sections (B, D, F, and H) of IMQ-induced psoriasis-like dermatitis mice in different treatment groups. (A–H) After treatment for 10 days. (A, B) sections of IMQ-induced psoriasis-like dermatitis dorsal skin and right ear pinna treated with acitretin showed greatly decreased inflammatory infiltration and hyperplasia of the epidermis. (C, D) WCR 800 mg/kg treated mice showed slightly reduced inflammatory infiltrates relative to IMQ-treated mice. (E, F) WCR 1,600 mg/kg treated group had significant decreases in thickened epidermis, hyperplasia, and inflammatory cell infiltration. (G, H) WCR 3,200 mg/kg treated group showed maximum efficacy of treatment with recovered tissue having normal epidermis, dermis, sebaceous glands, and hair follicles. (c) H&E stained tissue sections from the spleens of mice in different treatment groups. (A) A sectional view of a control group spleen showing normal white pulp and normal red pulp. (B) A sectional view of an IMQ-treated mouse spleen showing depletion of white pulp. (C–F) Sectional views of acitretin, WCR 800, 1,600, and 3,200 mg/kg treated mice, respectively, showing dose-dependent depletion of white pulp. Ctrl, control; IMQ, imiquimod; Gr, group; DS, dorsal skin; and EP, ear pinna.

Analysis of H&E stained sections from the IMQ-treated dorsal skin was found to be in line with the phenotypical observations and PASI score results. The dorsal skin and right ear pinna sections of the IMQ-treated mice showed significantly increased acanthosis, hyperkeratosis of the epidermis, and inflammatory infiltration (Figure 4(a)((C-D) and (G-H))). However, the dorsal skin and right ear pinna of control mice sections were normal in both epidermis and dermis (Figure 4(a)((A-B) and (E-F))). Interestingly, epidermal thickness was greatly reduced in the acitretin- and WCR-treated mice compared with the IMQ-treated mice (Figure 4(b)(A–H)). Mice treated with 3,200 mg/kg of WCR (group VI) showed effects similar to those of acitretin-treated mice (group III), where almost complete recovery from the IMQ-induced hyperplasia of the epidermal and subcutaneous tissue with only minor inflammatory reaction was observed. Mice in group V, administered with WCR at 1,600 mg/kg, were also found to exhibit ameliorative effects against IMQ-induced psoriasis-like dermatitis. However, mice in group IV (WCR 800 mg/kg treatment) had the lowest levels of antipsoriatic effects compared to other groups. The spleen sections of control mice showed normal red pulps and white pulps (Figure 4(c)(A)), whereas the spleen sections of IMQ-treated mice exhibited increased cellularity of the white pulp (T-cell-rich region) (Figure 4(c)(B)). In contrast, decreased cellularity of the white pulp region occurred after the administration of acitretin (5.14 mg/kg) and WCR (3,200 mg/kg) (Figure 4(c)(C–F)).

### 3.4. Effect of WCR Treatment on the Ratio of Spleen Weight to Bodyweight

**Figure 5:**
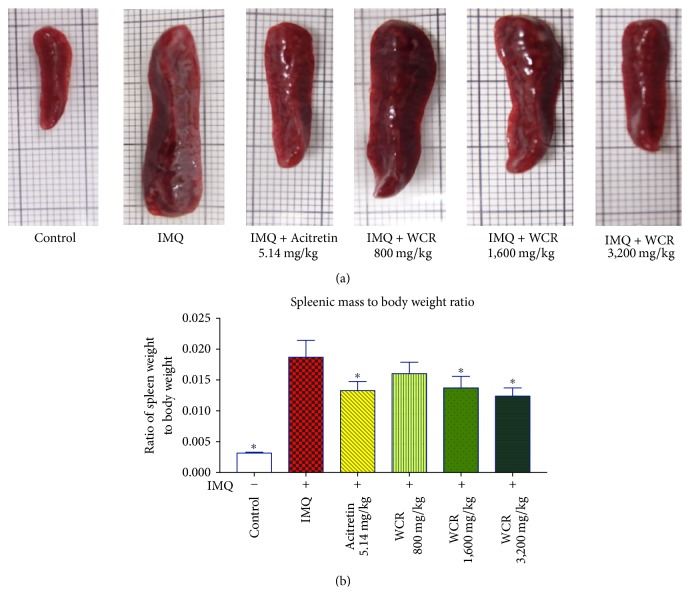
Effect of WCR treatment on the ratio of spleen weight to bodyweight. (a) Representative photographs of IMQ-induced mice: normal, untreated (IMQ alone), treated with acitretin (5.14 mg/kg), and WCR 800 mg/kg, WCR 1,600 mg/kg, or WCR 3,200 mg/kg. (b) At 24 h after the final administration, mice were sacrificed and the ratio of spleen weight to bodyweight was determined. Data presented are mean ± SD (*n* = 7) ^*∗*^*P* < 0.005 indicates a statistically significant difference from the IMQ group.

The size and weight of the spleen were markedly enlarged in IMQ-induced psoriasis mice [25]. The ratio of spleen weight to bodyweight was significantly decreased in the acitretin and WCR treatment group, even not to the level of the ratio of the control group (Figures 5(a) and 5(b)).

### 3.5. Effect of WCR Treatment on Increased Inflammatory Cytokines in Serum and Skin of IMQ-Induced Psoriasis-Like Dermatitis in Mice

**Figure 6:**
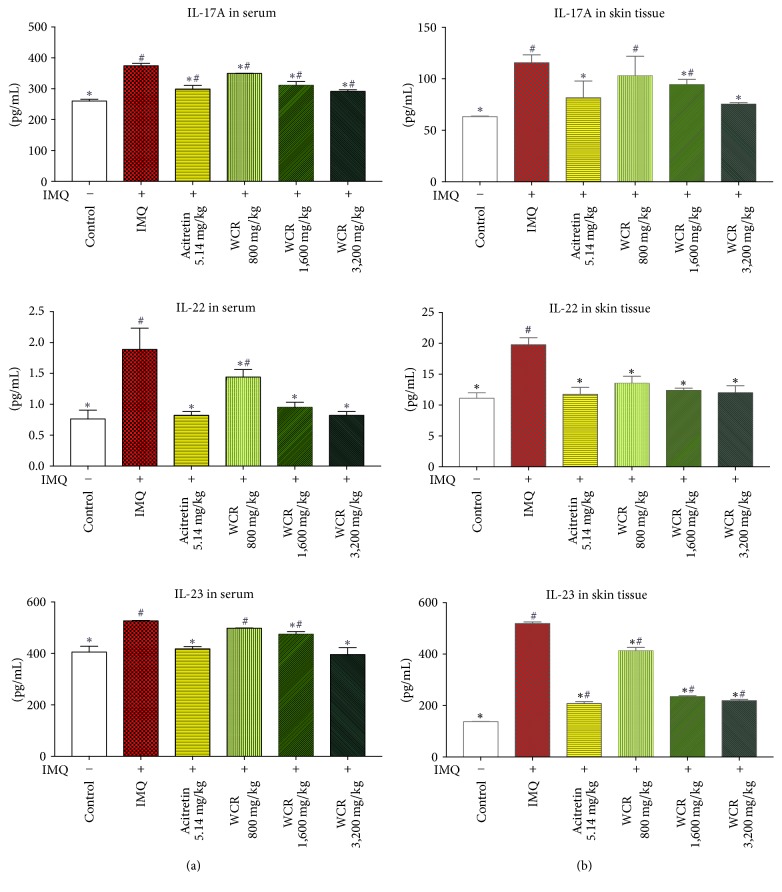
Effect of WCR treatment on serum and skin cytokine profiles. The concentrations of IL-17A, IL-22, and IL-23 in (a) the serum and (b) skin were measured using ELISA 24 h after the final treatment. Data presented as mean ± SD of 7 mice. ^*∗*^*P* < 0.05 indicates a statistically significant difference from the IMQ group. ^#^*P* < 0.05 indicates a statistically significant difference from the control group.

Inflammatory cytokines responsible for inflammation in psoriasis were measured in the serum and skin samples of normal, IMQ-treated mice and WCR-treated psoriasis-like dermatitis-induced mice by ELISA assay. Compared to the normal group, the levels of Th17-mediated cytokines, particularly IL-17A, IL-22, and IL-23, were significantly higher in both serum and dorsal skin samples from IMQ-treated mice. However, a marked dose-dependent reduction in the expression of IL-17A, IL-22, and IL-23 was noted in the WCR-treated groups compared to the IMQ-only treated group. There was no significant difference of IL-22 in skin samples between the treatment groups and the normal group (Figure 6).

## 4. Discussion

A combination of plant species (called a formulae) based on Thai traditional medicine is often prescribed by qualified Thai traditional doctors to enhance therapeutic efficacy and reduce side effects [26]. WCR, containing 8 different crude herbs, has been used to treat psoriasis in Thai patients at Thai Traditional Medicine Clinic of Prapokklao Hospital since 2006. Previous research has shown that WCR has a clinical effect of 93% on blood-heat type psoriasis [11]. Our clinical experience indicates that* A. galanga* and* S. glabra* are the principal components of the formula, while the other ingredients serve as pharmaceutical excipients. Evidence is accumulating that* A. galanga*, which contains terpenes and flavonoids, has strong antioxidant activity and significantly reduces the expression of the NF-*κ*B signaling biomarker [10] leading to a decrease of cytokine production by T-cells [27]. Astilbin, which is isolated from* S. glabra*, has significantly inhibitory effects on nitric oxide, TNF-*α*, and IL-6 production [28] through p65, ERK1/2, and JNK pathways [29]. In addition, anti-inflammatory, antiproliferative, and antioxidant activities have been well documented for* S. corbularia* [30],* R. nasutus *[31, 32],* A. ebracteatus *[33],* S. involute*,* S. collinsae* [34], and* Smilax* sp. [35]. As psoriasis is an immune-mediated inflammatory skin disorder, this extensive evidence of pharmacological activity may be indicative of the potential for using of WCR in treatment of psoriasis.

IMQ is an agonist of the toll-like receptor-7/8 (TLR-7/8) which has been approved for the treatment of actinic keratosis, external genital warts, and superficial basal cell carcinoma [13]. IMQ-induced psoriasis-like dermatitis in mice is mediated through the IL-23/IL-17 axis. This mouse model has been described as closely resembling human plaque-type psoriasis with respect to inflammatory infiltration, redness, thickening, and scaling of skin [14]. To treat psoriasis, established conventional systemic drugs such as methotrexate, cyclosporine, and acitretin have been the first line of treatment [4]. However, these agents seem to cause many serious adverse effects that some patients might be intolerant of during long-term treatment [6]. Therefore, there is a demand for new effective and safe therapeutic methods. One potential method involves traditional herbal medicines which have shown to be less costly than phototherapy and other new biologic agents [36]. The present study investigated whether a traditional Thai formula known as WCR could attenuate IMQ-induced psoriasis-like dermatitis in mice.

In this study, all the IMQ-treated mice developed prominent characteristics of psoriatic plaques such as erythema, skin thickening, scaling, parakeratosis, and acanthosis. Although these characteristics were clearly observable, our preliminary study found that the psoriasis-like symptoms tended to spontaneously decline after 6 days of IMQ treatment. These findings are consistent with recent research which has reported instability in the IMQ-induced psoriasis-like model due to the adaptive reaction of the mouse skin to IMQ stimulation [19]. The short-term inflammation of IMQ-induced psoriasis-like dermatitis in mice presents particular limitations. For that reason, we applied IMQ cream on the shaved dorsal skin of the mice for 15 consecutive days as described previously with some modifications [20].

We demonstrated that the IMQ-induced psoriasis-like dermatitis was noticeably improved after the treatment with WCR. Furthermore, a decrease in production of Th17-related cytokines including IL-17A, IL-22, and IL-23 in serum and skin tissue occurred in the WCR-treated mice. It has been well documented that Th17 is involved in many other processes with inflammatory and autoimmune diseases [37]. Th17 is maintained and developed by having IL-23 as a key cytokine in both murines and humans [38]. IL-23 is produced mainly by activated macrophages and dendritic cells, and increased expression of IL-23 is frequently found in psoriatic skin lesions [39]. Evidence has shown that keratinocyte proliferation and epidermal hyperplasia can be stimulated by IL-23 intradermal injection in psoriatic patients [40]. In psoriatic skin and systemic circulation, IL-17A, IL-22, and TNF-*α*, which are downstream effector molecules of Th17 pathway, are also presented at high levels [41]. These Th17-related cytokines further infiltrate keratinocytes, thereby amplifying local inflammation as well as causing keratinocyte hyperproliferation [5]. Our study revealed that inflammatory cytokines, including IL-17A, IL-22, and IL-23, in serum and dorsal skin tissue were reduced in WCR-treated mice. This finding was consistent with our previous study showing that WCR significantly inhibited the TNF-*α*- and IFN-*γ*-stimulated IL-17A, IL-22, and IL-23 secretion in HaCaT cells [12]. Although specific mechanisms and signaling pathways in the reduction of these cytokines are as yet undefined, we suspect that the antiproliferative and anti-inflammatory effect of WCR operates through the IL-23/Th17 axis.

The spleen is the largest secondary lymphoid organ in the immune system [42]. Twenty-four hours after the last treatment, the spleens of the mice were isolated and weight separately. Splenomegaly was induced by IMQ through systemic effects, whereas WCR significantly reduced the spleen weight to bodyweight ratio. That reduction was accompanied by a decrease in the cellularity of the periarteriolar lymphoid sheaths (PALS, T-cell area). Other causes such as irradiation, viruses, or drugs which result in necrosis or apoptosis of the T cells can also decrease cellularity of the PALS region [42]. Therefore, the decrease of the spleen weight/bodyweight ratio and cellularity of PALS after treatment with WCR in the IMQ-induced psoriasis-like dermatitis mice may be suggestive of a deficit in T-independent humoral immune responses.

Systemic treatment of psoriasis is amenable to immunosuppressants; however, they produce undesirable side effects and have serious toxicity profile. Acitretin, a retinoid derivative, offers the advantage of being a nonimmunosuppressive drug with a better safety profile. It exerts its effects on gene expression by activating two families of receptors—retinoic acid receptors (RARs) and retinoid X receptors (RXRs)—that are members of the steroid receptor superfamily [43]. Upon binding to a retinoid, those heterodimer receptors bind to specific DNA sequences called retinoic acid-responsive elements that activate transcription of genes whose products produce both the desirable pharmacological effects of acitretin but also their unwanted side effects [44]. Although acitretin causes less toxicity than immunosuppressants, its adverse side effects such as mucocutaneous adverse effects, teratogenic effects, hyperlipidemia, and elevation of serum liver enzymes could influence compliance of patients [45]. In the present study, acitretin was used as a standard reference drug for comparison with the antipsoriatic effects of WCR. The results showed that there was no significant difference in the levels of inflammatory cytokines, phenotypical observations, histopathological findings, or size of spleen between acitretin-treated and WCR- treated mice. This finding is promising that WCR has a pivotal place in the group of therapeutic alternatives for treatment of psoriasis. However, further investigation into molecular mechanisms of action in all aspects is required to verify that WCR can be effectively and specifically used to control psoriasis in patients.

## 5. Conclusion

The present study demonstrates that WCR exhibits promising potential for attenuating inflammatory and psoriatic symptoms underlying psoriasis-like dermatitis in mice. This preclinical evidence suggests that WCR may have the pharmacological actions of regulating Th17 related cytokines, making it potentially useful as an alternative therapeutic strategy for psoriasis. Further investigation is needed to evaluate acute and chronic toxicities in animal models prior to performing a clinical study.

## Figures and Tables

**Table 1:** Plants used in WCR and their original sources.

	Thai name	Scientific name	Family name	Part used	Original source
(1)	Kha	*Alpinia galanga* (L.) Willd.	Zingiberaceae	Rhizomes	Chanthaburi Province, Thailand
(2)	Khao Yen Tai	*Smilax glabra* Wall.ex Roxb.	Smilacaceae	Rhizomes	
(3)	Khao Yen Nuea	*Smilax corbularia* Kunth.	Smilacaceae	Rhizomes	
(4)	Khao Yen Jeen	*Smilax *sp.	Smilacaceae	Rhizomes	

(5)	Hua Ta Pead	*Stemona involuta* Inthachub.	Stemonaceae	Roots	Chachoengsao Province, Thailand
(6)	Non Tai Yak	*Stemona collinsae *Craib.	Stemonaceae	Roots	

(7)	Thong Pan Chang	*Rhinacanthus nasutus* (L.) Kurz.	Acanthaceae	Aerial part	Chanthaburi Province, Thailand
(8)	Ngueak plaamo	*Acanthus ilicifolius *L.	Acanthaceae	Aerial part	

## Additional Points

*Availability of Data and Materials*. We have no special datasets in this article, but our data is available for reference.

## References

[1] Schön M. P., Boehncke W.-H. (2005). Psoriasis. *The New England Journal of Medicine*.

[2] Dubertret L., Mrowietz U., Ranki A. (2006). European patient perspectives on the impact of psoriasis: The EUROPSO patient membership survey. *British Journal of Dermatology*.

[3] Griffiths C. E., Barker J. N. (2007). Pathogenesis and clinical features of psoriasis. *The Lancet*.

[4] Boehncke W.-H., Schön M. P. (2015). Psoriasis. *The Lancet*.

[5] Cai Y., Fleming C., Yan J. (2012). New insights of T cells in the pathogenesis of psoriasis. *Cellular & Molecular Immunology*.

[6] Menter A., Korman N. J., Elmets C. A. (2009). Guidelines of care for the management of psoriasis and psoriatic arthritis. Section 4. Guidelines of care for the management and treatment of psoriasis with traditional systemic agents. *Journal of the American Academy of Dermatology*.

[7] Menter A., Gottlieb A., Feldman S. R. (2008). Guidelines of care for the management of psoriasis and psoriatic arthritis. Section 1. Overview of psoriasis and guidelines of care for the treatment of psoriasis with biologics. *Journal of the American Academy of Dermatology*.

[8] Augustin M., Holland B., Dartsch D., Langenbruch A., Radtke M. A. (2011). Adherence in the treatment of psoriasis: A systematic review. *Dermatology*.

[9] Corson T. W., Crews C. M. (2007). Molecular understanding and modern application of traditional medicines: triumphs and trials. *Cell*.

[10] Saelee C., Thongrakard V., Tencomnao T. (2011). Effects of thai medicinal herb extracts with anti-psoriatic activity on the expression on NF-*κ*b signaling biomarkers in hacat keratinocytes. *Molecules*.

[11] Swaddichai C., Sukpaisarn V., Chaichareonpong K., Sanguantrap P., Limprapaipong T. (2011). Monitoring of psoriasis patients in thai traditional medicine clinic of prapokklao hospital. *Journal of Thai Traditional & Alternative Medicine*.

[12] Na Takuathung M., Wongnoppavich A., Pitchakarn P. (2017). Effects of wannachawee recipe with antipsoriatic activity on suppressing inflammatory cytokine production in hacat human keratinocytes. *Evidence-Based Complementary and Alternative Medicine*.

[13] Flutter B., Nestle F. O. (2013). TLRs to cytokines: mechanistic insights from the imiquimod mouse model of psoriasis. *European Journal of Immunology*.

[14] van der Fits L., Mourits S., Voerman J. S. A. (2009). Imiquimod-induced psoriasis-like skin inflammation in mice is mediated via the IL-23/IL-17 axis. *The Journal of Immunology*.

[15] World Health Organization (2007). *WHO Guidelines for Assessing Quality of Herbal Medicines with Reference to Contaminants and Residues*.

[16] Ministry of Public Health. Thai Herbal Pharmacopoeia Volume IV., Bangkok, Department of Medical Sciences, 2014

[17] Faul F., Erdfelder E., Lang A., Buchner A. (2007). G^*^Power 3: a flexible statistical power analysis program for the social, behavioral, and biomedical sciences. *Behavior Research Methods*.

[18] Faul F., Erdfelder E., Buchner A., Lang A.-G. (2009). Statistical power analyses using G∗Power 3.1: tests for correlation and regression analyses. *Behavior Research Methods*.

[19] Sun J., Zhao Y., Hu J. (2013). Curcumin inhibits imiquimod-induced psoriasis-like inflammation by inhibiting IL-1beta and IL-6 production in mice. *PLoS ONE*.

[20] Kim C.-H., Kim J.-Y., Lee A.-Y. (2015). Therapeutic and immunomodulatory effects of glucosamine in combination with low-dose cyclosporine A in a murine model of imiquimod-induced psoriasis. *European Journal of Pharmacology*.

[21] Muruganantham N., Basavaraj K. H., Dhanabal S. P., Praveen T. K., Shamasundar N. M., Rao K. S. (2011). Screening of Caesalpinia bonduc leaves for antipsoriatic activity. *Journal of Ethnopharmacology*.

[22] Christensen S. C., Johnson T. E., Markel P. D. (1996). Quantitative trait locus analyses of sleep-times induced by sedative-hypnotics in LSXSS recombinant inbred strains of mice. *Alcoholism: Clinical and Experimental Research*.

[23] Arora N., Shah K., Pandey-Rai S. (2016). Inhibition of imiquimod-induced psoriasis-like dermatitis in mice by herbal extracts from some Indian medicinal plants. *Protoplasma*.

[24] Grine L., Steeland S., Van Ryckeghem S. (2016). Topical imiquimod yields systemic effects due to unintended oral uptake. *Scientific Reports*.

[25] Byamba D., Kim D. Y., Kim D.-S. (2014). Skin-penetrating methotrexate alleviates imiquimod-induced psoriasiform dermatitis via decreasing IL-17-producing gamma delta T cells. *Experimental Dermatology*.

[26] Disayavanish C., Disayavanish P. (1998). Introduction of the treatment method of Thai traditional medicine: Its validity and future perspectives. *Psychiatry and Clinical Neurosciences*.

[27] Chudiwal A. K., Jain D. P., Somani R. S. (2010). Alpinia galanga Willd.- An overview on phyto-pharmacological properties. *Indian Journal of Natural Products and Resources (IJNPR)*.

[28] Lu C.-L., Zhu W., Wang D.-M. (2015). Inhibitory effects of chemical compounds isolated from the rhizome of smilax glabra on nitric oxide and tumor necrosis factor-*α* production in lipopolysaccharide-induced RAW264.7 cell. *Evidence-Based Complementary and Alternative Medicine*.

[29] Lu C.-L., Zhu Y.-F., Hu M.-M. (2015). Optimization of astilbin extraction from the rhizome of smilax glabra, and evaluation of its anti-inflammatory effect and probable underlying mechanism in lipopolysaccharide-induced raw264.7 macrophages. *Molecules*.

[30] Reanmongkol W., Itharat A., Bouking P. (2007). Evaluation of the anti-inflammatory, antinociceptive and antipyretic activities of the extracts from Smilax corbularia Kunth rhizomes in mice and rats (in vivo). *Songklanakarin Journal of Science and Technology*.

[31] Bukke S., Raghu P. S., Sailaja G., Kedam T. R. (2011). The study on morphological, phytochemical and pharmacological aspects of Rhinacanthus nasutus. (L) kurz (A review). *Journal of Applied Pharmaceutical Science*.

[32] Gotoh A., Sakaeda T., Kimura T. (2004). Antiproliferative activity of Rhinacanthus nasutus (L.) KURZ extracts and the active moiety, rhinacanthin C. *Biological & Pharmaceutical Bulletin*.

[33] Singh D., Aeri V. (2013). Phytochemical and pharmacological potential of Acanthus ilicifolius. *Journal of Pharmacy and Bioallied Sciences*.

[34] Brem B., Seger C., Pacher T. (2004). Antioxidant dehydrotocopherols as a new chemical character of Stemona species. *Phytochemistry*.

[35] Vijayalakshmi A., Ravichandiran V., Velraj M., Nirmala S., Jayakumari S. (2012). Screening of flavonoid "quercetin" from the rhizome of Smilax china Linn. for anti-psoriatic activity. *Asian Pacific Journal of Tropical Biomedicine*.

[36] Wang G., Liu Y. (2004). Traditional Chinese medicine is effective and safe in the treatment of psoriasis. *International Journal of Dermatology*.

[37] Korn T., Bettelli E., Oukka M., Kuchroo V. K. (2009). IL-17 and Th17 cells. *Annual Review of Immunology*.

[38] Boniface K., Blom B., Liu Y.-J., De Waal Malefyt R. (2008). From interleukin-23 to T-helper 17 cells: Human T-helper cell differentiation revisited. *Immunological Reviews*.

[39] Lee E., Trepicchio W. L., Oestreicher J. L. (2004). Increased expression of interleukin 23 p19 and p40 in lesional skin of patients with psoriasis vulgaris. *The Journal of Experimental Medicine*.

[40] Chan J. R., Blumenschein W., Murphy E. (2006). IL-23 stimulates epidermal hyperplasia via TNF and IL-20R2-dependent mechanisms with implications for psoriasis pathogenesis. *The Journal of Experimental Medicine*.

[41] Van Belle A. B., De Heusch M., Lemaire M. M. (2012). IL-22 is required for imiquimod-induced psoriasiform skin inflammation in mice. *The Journal of Immunology*.

[42] Elmore S. A. (2006). Enhanced Histopathology of the Spleen. *Toxicologic Pathology*.

[43] Winterfield L., Cather J., Cather J., Menter A. (2003). Changing paradigms in dermatology: Nuclear hormone receptors. *Clinics in Dermatology*.

[44] Burkhart C., Morrell D., Goldsmith L., Brunton L. L., Chabner B. A., Knollmann C. B. (2011). Goodman & Gilman's the pharmacological basis of therapeutics.

[45] Dogra S., Yadav S. (2014). Acitretin in psoriasis: An evolving scenario. *International Journal of Dermatology*.

